# Nck1 activity in lateral amygdala regulates long-term fear memory formation

**DOI:** 10.1038/s41398-022-02244-x

**Published:** 2022-11-12

**Authors:** Or Ilovich, Monica Dines, Blesson K. Paul, Edi Barkai, Raphael Lamprecht

**Affiliations:** Sagol Department of Neurobiology, Faculty of Natural Sciences, University of Haifa, Haifa, Israel

**Keywords:** Long-term memory, Molecular neuroscience

## Abstract

Fear conditioning leads to long-term fear memory formation and is a model for studying fear-related psychopathological conditions such as phobias and post-traumatic stress disorder. Long-term fear memory formation is believed to involve alterations of synaptic efficacy mediated by changes in synaptic transmission and morphology in lateral amygdala (LA). Nck1 is a key neuronal adaptor protein involved in the regulation of the actin cytoskeleton and the neuronal processes believed to be involved in memory formation. However, the role of Nck1 in memory formation is not known. Here we explored the role of Nck1 in fear memory formation in lateral amygdala (LA). Reduction of Nck1 in excitatory neurons in LA enhanced long-term, but not short-term, auditory fear conditioning memory. Activation of Nck1, by using a photoactivatable Nck1 (PA-Nck1), during auditory fear conditioning in excitatory neurons in LA impaired long-term, but not short-term, fear memory. Activation of Nck1 immediately or a day after fear conditioning did not affect fear memory. The hippocampal-mediated contextual fear memory was not affected by the reduction or activation of Nck1 in LA. We show that Nck1 is localized to the presynapses in LA. Nck1 activation in LA excitatory neurons decreased the frequency of AMPA receptors-mediated miniature excitatory synaptic currents (mEPSCs). Nck1 activation did not affect GABA receptor-mediated inhibitory synaptic currents (mIPSCs). These results show that Nck1 activity in excitatory neurons in LA regulates glutamate release and sets the threshold for fear memory formation. Moreover, our research shows that Nck1 may serve as a target for pharmacological treatment of fear and anxiety disorders.

## Introduction

Alterations of fear have a significant role in stress and anxiety disorders in humans [1, 2]. While there are several experimental tools for studying fear and anxiety, one of the most straightforward is fear conditioning [3, 4]. In fear conditioning, an animal associates a neutral stimulus, such as a tone, with an aversive event, typically a mild footshock [5–9]. This paradigm is especially useful as a tool for studying the molecular basis of long-term fear memory because a putative site of memory, the lateral nucleus of the amygdala (LA), has been identified [5–11]. Thus, fear conditioning provides a behavioral tool and anatomical site to assess molecular mechanisms that might mediate changes in synaptic efficacy during long-term fear memory formation and fear-related disorders, such as post-traumatic stress disorder and phobias.

Long-term memory (LTM) formation is believed to involve alterations of synaptic efficacy produced by modifications in neural transmission caused by chemical and/or structural modifications of synaptic communication within neuronal networks [12–17]. However, the cellular and molecular mechanisms of synaptic plasticity that underlie memory formation and storage are not well understood. Actin is a most attractive candidate to play a key role in memory formation as it is responsive to synaptic signaling, such as triggered during learning, and consequently may mediate cellular events that underlie changes in synaptic efficacy, such as synaptic transmission and morphology [18–20]. On the other hand, candidate genes for psychiatric and neurodevelopmental disorders encode actin cytoskeleton-regulatory proteins indicating that imbalanced regulation of the actin cytoskeleton may cause cognitive and mental dysfunctions [21].

Nck1 is a key neuronal adaptor molecule that is involved in mediating between synaptic molecular activity, actin cytoskeleton and neuronal functions and therefore may be involved in memory formation in adults. The Nck family of SH2/SH3 domains-containing adaptors consists of Nck1/α and Nck2/β [22, 23]. Nck is comprised of one SH2 and three SH3 domains and has a key role in pTyr signaling from the cell surface to regulate the actin cytoskeleton [24]. Studies have shown that Nck can exert its effect by clustering [25, 26] and that such clustering leads to actin polymerization [26–29].

Nck1 is expressed in the brain and implicated in brain psychiatric disorders and other brain dysfunctions such as in schizophrenia and neuroticism [30–33] however its role in memory formation is not known. We were interested to examine whether Nck1 activity in excitatory neurons in LA affects fear memory formation. To examine the roles of Nck1 in fear memory formation we inhibited Nck1 (by shRNA) or activated it (by light using Nck1 photoactivatable construct (CRY2olig–mCh–Nck1 [34])) in excitatory neurons in LA (by expressing the constructs downstream to the CaMKII promoter [35]). We revealed that decreasing Nck1 activity in LA excitatory neurons facilitates auditory long-term fear memory formation whereas increasing Nck1 activity in LA neurons impairs long-term fear memory and presynaptic release of glutamate. Thus, Nck1 activity in LA suppresses presynaptic glutamate release and long-term fear memory formation.

## Materials and methods

### Animals

Male C57BL6 mice (12–20 weeks of age) were used in this study (Harlan Laboratories). Following surgery, mice were housed separately at 22 ± 2 °C in a 12 h light/dark cycle, with ad libitum access to food and water. All experiments were done following the instructions and approval of the University of Haifa animal ethics committee for animal experiments observing National Institutes of Health guidelines and all experiments were performed following the relevant guidelines and regulations.

### AAVs production

shRNAs containing AAV was generated by the Viral Vector Facility VVF, Institute of Pharmacology and Toxicology, University of Zurich. BLOCK-iT™ RNAi Designer (Invitrogen, Thermo Fisher Scientific) was used to identify 21-mer short/small hairpin (sh) RNA sequences that are predicted to downregulate mouse Nck1. The following four shRNAs sh(mNck1) were selected against the mouse Nck1 transcript NM_010878.3: 5’-GCTGGATATCAAGAAGAATGA-3’; 5’-GCGATGTAATGGATGTTATTG-3’; 5’-GGAGGAACTTGTAGAACATTA-3’; 5’-GCTTTAACTGGTCATGTAACT-3’. A human microRNA-30-embedded shRNA sequences (miR-E) construct was generated (pssAAV-2-mCaMKIIa(short)-chI[4xsh(mNck1)])-EGFP-WPRE-bGHp(A)) [36] (titer: 9.3X10E12VG/ml). Control shRNAs are four identical (4x) human microRNA-30-based short/small hairpin (sh) non-targeting (NT) RNAs ssAAV-2-mCaMKIIα(short)-chI[4x(shm/rNS)]-EGFP-WPRE-bGHp(A) (titer: 1X10E13VG/ml).

We used rAAV2/1 vectors containing short CaMKII promoter sCKII-Cry2Olig-mCherry-Nck1 (titer: 2.24E + 13VG/ml, ELSC Vector Core Facility, The Hebrew University of Jerusalem, Israel). CRY2olig-mCh-Nck was a gift from Chandra Tucker (Addgene plasmid # 60034; http://n2t.net/addgene:60034; RRID: Addgene_60034).

### AAV injection

Mice were anesthetized with Dormitor (1 mg/ml in PBS) and Ketamine (100 mg/ml in PBS) cocktail at 10 µl/g mouse weight. The virus was injected intracranially into each hemisphere (0.5 µl/hemisphere, 0.1 µl/min) aiming at the lateral amygdala (LA) at the following coordinates: anterior-posterior −1.4, medial-lateral ±3.3, dorsal-ventral 4.65 (relative to Bregma). In optogenetic experiments, after virus injection, intracranial optic fiber (Thorlabs, Fiber Optic Cannula, Ø1.25 mm Stainless Ferrule, Ø200 µm Core, 0.39 NA) was implanted on the same line and 0.5 mm above the virus injection coordinates. Animals were allowed to recuperate for 4 weeks before behavioral experiments.

### Fear conditioning

On the day of training, mice were placed in a training chamber (Coulbourn Instruments). Mice were allowed to acclimate in the chamber for 2 min and then subjected to 3 pairs of tone (Conditioned stimulus (CS)—20 s, 2.8 kHz, 85 dB) that co-terminated with a foot shock (Unconditioned stimulus (US)—2 s, 0.8 mA). The inter-trial interval was 120 s. Mice were tested for contextual fear conditioning in the same context (for 9 min) 1 h after training for short-term memory or 24 h after training for long-term memory. Mice were tested for auditory fear conditioning in a different context 2 h after training for short-term memory or 48 h after training for long-term memory. Behavior was recorded and the video images were transferred to a computer equipped with an analysis program. The percentage of changed pixels between two adjacent 0.25 s images was used as a measure of activity.

### Light stimulation

Blue light (473 nm) from a laser was subjected at the indicated time (see below) to activate PA-Nck1. Optic fibers were connected to a 473-nm blue laser diode (Shanghai Dreamlasers) via an FC/PC adaptor. The light intensity ~15 mW/mm^2^ was measured at the tip of the fiber. A control group of animals got an equal amount of virus microinjection into LA/BLA along with the fiber optic implantation but did not receive light stimulation.

### Transfection, light stimulation and phalloidin staining of HEK293 cells

HEK293 (Human Embryonic Kidney 293T) cells were grown in cell culture dishes (Cellstar cell culture dishes, PS, 100 × 20 mm, with vents, sterile) with 0.5 ml of complete Dulbecco’s modified Eagle’s medium (DMEM) (5% heat-inactivated bovine serum, 100 units/ml penicillin and 100 μg/ml streptomycin) at a 37 °C inside an incubator with humidified air containing 5% CO_2_. Coverslips were inserted into 24 wells plate (costar 24 wells cell culture plate by corning incorporated) before adding the cells and medium. Cells were incubated for 24 h to allow the cells to adhere to the coverslip, forming a monolayer of 70–80% confluency at the transfection.

The plasmid, CRY2olig-mCh-Nck (Addgene, Plasmid #60034), was transfected according to the PolyJet transfection kit protocol. The DMEM complete medium was replaced by fresh DMEM complete media 30 min before transfection. Then we added a PolyJet (1.5 μl) and DNA (500 ng) diluted in a total of 50 μl serum-free DMEM medium with high glucose, and incubated at room temperature for 13 min to allow the transfection complex to be formed. Then the mix was added to each well of the plate. After incubation of 24 h, the success of the transfection was visualized by a fluorescent microscope.

The transfected HEK293 cells with CRY2olig-mCh-Nck plasmid were exposed to blue light (488 nm) for 3 s every 3 min for 30 min. Immediately afterward the cells were fixated with 4% PFA diluted in PBS. The cells were washed 3 times with PBS. For staining the cells were permeabilized by 0.5% Triton x-100 diluted in PBS and stained with acti-stain 488-phalloidin (0.0973 μM, Cytoskeleton, Inc.) for 30 min. The cells were washed three times with PBS. After staining the cells were imaged using a confocal microscope.

### Histology and phalloidin staining of amygdala slices

After behavioral testing, the mice injected with AAV were perfused with 3.7% PFA in PBS and their brains were placed in 30% sucrose in PBS at 4^o^C for 48 h for post-fixation and then they were frozen. The brains were sliced at a thickness of 45 μm using a cryostat. Slices were tested for expression of mCherry in the brain. Only mice with expression of mCherry in LA/BLA were included in the data analysis. For analysis of PA-Nck1 clusters, the animals were exposed to light in one hemisphere (3 light stimulating of 20 s with 2 min intervals) 48 h after the last test (auditory cued test). After light stimulation, the mice were perfused and the brains were removed, post-fixed and sliced as above. For phalloidin staining, after stimulation, the fixation and slicing were as above. The slices were washed 3 times with PBS. Slices were blocked in 10% NGS and permeabilized by 0.5% Triton x-100 diluted in PBS for 10 min. The slices were subjected to Acti-stain 488 phalloidin (0.0973 μM, Cytoskeleton, Inc.) for 45 min. The brain slices were mounted on slides and imaged. Analyses of PA-Nck1 and F-actin clusters in light-activated LA and LA in dark were performed.

### Image analysis

Cells were photographed using a Nikon confocal microscope. The images were analyzed using ImageJ (Fiji), with the FindFoci GUI plugin. Eight-bit mono photos were analyzed using the Otsu method for thresholding. Region of interest (ROI) for PA-Nck1 was selected (30 × 30 and 50 × 50 pixels for HEK and amygdala cells, respectively). The number of clusters of F-actin is calculated for the whole cell body. The number of clusters detected above the threshold is divided by average intensity across the cell (for mCherry in the case of Nck1 and Phalloidin for actin) for normalization.

### Western blot preparation and analysis

The mice were anesthetized using isoflurane, decapitated and their brains were extracted, frozen immediately by dry ice and stored at −80 °C. The amygdala was dissected. Fifty µl of lysis buffer (10% Glycerol, 1% Triton X-100, 1 mM EDTA, 50 nM HEPES, 150 nM NaCl) was added to the Eppendorf tube. The tissue was homogenized by a mashing stick. Then, the tubes were centrifuged for 5 min (12,000 rcf) and the pallet was discarded. Fifty µl of Laemmli sample buffer was added to the supernatant in the Eppendorf tube. Then the tubes were heated to 80 °C for 5 min. The samples were subjected to 12.5% SDS-PAGE with the Mini-PROTEAN system (Bio-Rad). After electrophoresis, the gels were transferred to the nitrocellulose membrane using the Mini-PROTEAN system (Bio-Rad). The membrane was blocked with 5% Difco Skim-Milk (232100, BD) and 0.3% Triton X-100 (93443, Sigma) in TBST (0010001885, bio-labs) for 30 min at RT. After blocking, anti-Nck1 (2319, Cell-signaling) and anti-EphA4 (sc-921, Santa Cruz) were added to the blocking solution at 1:1000 dilutions and left overnight at 4 °C. After incubation, the nitrocellulose membrane was washed in TBST (3 times × 5 min) and then incubated in HRP antibodies corresponding to the primary antibodies (1:10,000) dilutions in TBST for 1 h at RT. The nitrocellulose membrane was washed in TBST (3 times × 5 min) and EZ-ECL (Bio-Labs) was added and imaged at auto exposure (Amersham Imager, GE). Nck1 level was normalized to EphA4 levels.

### Immunohistochemistry

Mice were perfused as above and brains were sectioned into 45 µm thick slices using a cryostat (Leica CM1900) and stored in PBS. The slices were blocked in 5% NGS with 0.3% Triton X-100 (93443, Sigma) in TBST (0010001885, bio-labs) for 30 min. The slices were then incubated overnight at 4 °C in 1:500 dilution of anti-Nck1 (2319, Cell-signaling) and anti-Bassoon (SAP7F407, Enzo life sciences) or anti-PSD95 (sc-6926, Santa Cruz Biotechnology) in TBST. The next day, the slices were washed with TBST (3 times × 5 min) and then incubated at RT for 2 h in secondary antibodies (1:1000; Alexa 488 anti-mouse for anti-Bassoon, Alexa 568 anti-rabbit for anti-Nck1 or Alexa anti-goat 488 for anti-PSD95). After incubation, the slices were washed with TBST (3 times × 5 min) and then mounted on slides. Slides were visualized on a Nikon confocal microscope or Zeiss Elyra 7 super-resolution microscope at the SIM mode.

### Electrophysiology

#### Slice preparation

Brains were harvested by decapitation and 300 µm thick coronal brain slices were obtained in ice-cold oxygenated (95% O_2_–5% CO_2_) normal saline Ringer solution (in mM: 124 NaCl, 3 KCl, 2 MgSO_4_, 1.25 NaH_2_PO_4_, 26 NaHCO_3_, 2 CaCl_2_, and 10 glucose). Brains were hemisected, and around three slices per hemisphere containing lateral amygdala (LA) sections were incubated for at least 1 h at room temperature. Then, the slices were perfused with Ringer solution at 30 °C in a recording chamber located beneath an infrared DIC microscope (BX51WI Olympus; Tokyo, Japan). mCherry expressing pyramidal neurons in the LA were identified and visualized, and whole-cell voltage-clamp recordings were performed using Axopatch 200B amplifier (Molecular Devices, Sunnyvale, CA). Data were obtained utilizing pClamp9 (Molecular Devices) acquired at 2 kHz and digitized at 5 kHz, and the data were analyzed using miniAnalysis software.

#### Light stimulation

The slices that have a prominent LA region were chosen. After breaking the seal in whole-cell voltage-clamp mode the following blue light (473 nm) stimulation protocol was used. Before shining the light pulses, a period of 5 min was recorded for the baseline. A 20-s pulse followed by a 2 min inter-trial interval (ITI) was given with three repetitions.

#### AMPA-mediated miniature EPSC recording

To record AMPA (α-amino-3-hydroxy-5-methyl-4-isoxazolepropionic acid)-mediated mEPSCs, the cells were maintained at the physiological condition, held at resting potential of −80 mV. In these conditions, the NMDA receptors are rarely open, and most voltage-gated channels are closed. The recording electrode contained the following solution (in mM): 140 K-gluconate, 1 EGTA, 6 KCl, 4 NaCl, 2MgCl_2_, and 10 HEPES, pH 7.25, 280 mOsm. The recording was done in the presence of 1 μM tetrodotoxin.

#### GABAA-mediated miniature IPSC recording

To record GABAA-mediated miniature IPSCs (mIPSCs), the recording electrode containing the following solution was used (in mM): 140 cesium chloride, 1 EGTA, 6 KCl, 4 NaCl, 2 MgCl_2_, and 10 HEPES at pH 7.25, and 280 mOsm. In these conditions, the reversal potential of chloride is 0 mV, and thus strong GABAA-mediated currents can be studied at a holding potential of −60 mV. To the perfusion solution, TTX (1 µM) and DNQX (20 µM) to block AMPA receptors and APV (50 µM) to block NMDA receptors were added, thus allowing the recording of pure IPSCs.

### Statistics

We choose the sample size according to the acceptable in previous literature. Mice of the same age were randomly assigned to each group. The outcome assessment and data analysis were done blindly. Data were analyzed with repeated measures ANOVA for behavioral analysis and electrophysiology (with LSD post hoc analysis) and with *t*-test for behavioral studies (two groups) and PA-Nck1 and F-actin clustering studies with an α level of 0.05 using the PASW statistics 25.

## Results

### Reduction of Nck1 protein level in lateral amygdala enhances long-term auditory, but not contextual, fear conditioning memory

**Fig. 1:**
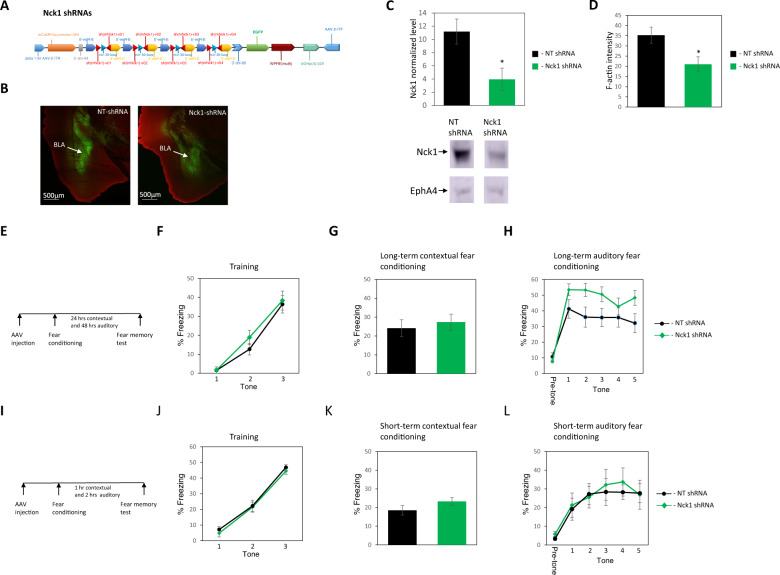
Reduction of Nck1 in excitatory neurons in lateral amygdala impairs long-term memory formation. **A** 21-mer short/small hairpin shRNA sequences that are selected specifically against the mouse Nck1 and are embedded in a human microRNA-30 (miR-E) were used in the study. **B** Nck1 shRNAs or control non-targeting (NT) shRNAs are expressed in the basolateral amygdala (BLA) as shown by the EGFP that is co-expressed with the shRNAs. **C** Nck1 shRNAs (*n* = 3) significantly reduces the level of Nck1 in the lateral amygdala when compared to NT shRNAs control (*n* = 3) (*p* = 0.048). A representative blot is shown. **D** F-actin level is reduced in Nck1 shRNA expressing neurons when compared to F-actin level in neurons expressing NT shRNA (*t*_(10)_ = 2.517, *p* = 0.031). **E** Description of the long-term behavioral protocol. **F** Freezing during fear conditioning training is not different between the Nck1 shRNA and NT shRNAs groups (*F*_(1,19)_ = 0.556, *p* = 0.456). **G** Freezing during the long-term contextual fear memory test is not different between the Nck1 shRNAs and NT shRNAs groups (*t*_(0.523)_ = 0.19, *p* = 0.607). **H** Reduced Nck1 in LA (*n* = 10) enhanced long-term auditory fear memory formation in LA when compared to the control group (*n* = 11) (*F*_(1,19)_ = 4.492, *p* = 0.047). **I** Description of the short-term behavioral protocol. **J** Freezing throughout the tones presentations during training of animals used in the STM experiment that expressed Nck1 shRNAs or control NT shRNAs was analyzed. There is no significant difference between the groups (*F*_(1,12)_ = 0.576, *p* = 0.462). **K** Contextual fear conditioning tested 1 h after fear conditioning is not different between the Nck1 shRNAs (*n* = 7) and control NT shRNAs (*n* = 7) groups (*t*_(12)_ = 1.456, *p* = 0.171). **L** Auditory fear conditioning tested 2 h after fear conditioning is not different between animals injected with shRNAs against Nck1 compared to NT shRNAs injected mice (*F*_(1,12)_ = 0.077, *p* = 0.786). Values are mean ± SEM. **C**, **D** Independent samples *t*-test; **F**–**H** and **J**–**L** Repeated measures ANOVA.

To examine the roles of Nck1 in memory formation we designed 21-mer short/small hairpin shRNA sequences that are selected specifically against the mouse Nck1 and are embedded in a human microRNA-30 (miR-E; Fig. 1A) and expressed them in excitatory neurons in BLA (Fig. 1B). The shRNAs significantly reduced the level of Nck1 protein in neurons expressing the shRNAs compared to neurons that expressed the control non-targeting (NT) shRNA 5 weeks after microinjection (*t*_(4)_ = 2.819, *p* = 0.048) (Fig. 1C). We also examined the content of F-actin in Nck1 shRNA (*n* = 5) and found that it is significantly reduced compared to NT shRNA (*n* = 7) (*t*_(10)_ = 2.517, *p* = 0.031) (Fig. 1D). Reducing Nck1 levels in LA had no effect on fear conditioning training (*F*_(1,19)_ = 0.556, *p* = 0.456) with no treatment × tone trial interaction (*F*_(1.508,28.653)_ = 0.657, *p* = 0.524) (Fig. 1F). Lower levels of Nck1 in LA had no effect on long-term contextual fear conditioning memory formation (*t*_(0.523)_ = 0.19, *p* = 0.607) (Fig. 1G). Reduced Nck1 in LA (*n* = 10) enhanced long-term fear memory formation when compared to the control group (*n* = 11) (*F*_(1,19)_ = 4.492; *p* = 0.047) (Fig. 1H). There is no treatment × tone trial interaction (*F*_(4,76)_ = 0.672, *p* = 0.613). Thus, Nck1 in LA constrains the formation of long-term fear memory and its removal enhances fear memory.

### Reduction of Nck1 protein in lateral amygdala does not affect short-term auditory and contextual fear conditioning memories

To test whether Nck1 is involved in controlling the ability to associate the CS and US or specifically suppresses long-term memory formation we tested the effects of Nck1 reduction on short-term fear conditioning memory. Animals were injected with shRNA against Nck1 or NT shRNA into their lateral amygdala. A month later they were trained for fear conditioning and tested 1 h afterward in the same context for contextual fear conditioning STM and 2 h later in a different context for auditory fear conditioning STM. There is no difference in freezing during training between the groups (*F*_(1,12)_ = 0.576, *p* = 0.462) and no treatment × tone trial interaction (*F*_(2,24)_ = 0.08, *p* = 0.923) (Fig. 1J). There is no difference in contextual fear conditioning STM between the groups (*t*_(12)_ = 1.456, *p* = 0.171) (Fig. 1K). Mice that were injected with shRNA against Nck1 (*n* = 7) were not different from mice injected with NT shRNA (*n* = 7) in short-term auditory fear conditioning memory (*F*_(1,12)_ = 0.077, *p* = 0.786) (Fig. 1L). There is no difference in treatment × tone trial interaction (*F*_(4,48)_ = 0.176, *p* = 0.950). We, therefore, conclude that Nck1 is not involved in inhibiting STM and the ability to form the association of CS and US but rather specifically in restricting the formation of long-term memory.

### Activation of Nck1 in lateral amygdala impairs long-term auditory fear conditioning memory

**Fig. 2:**
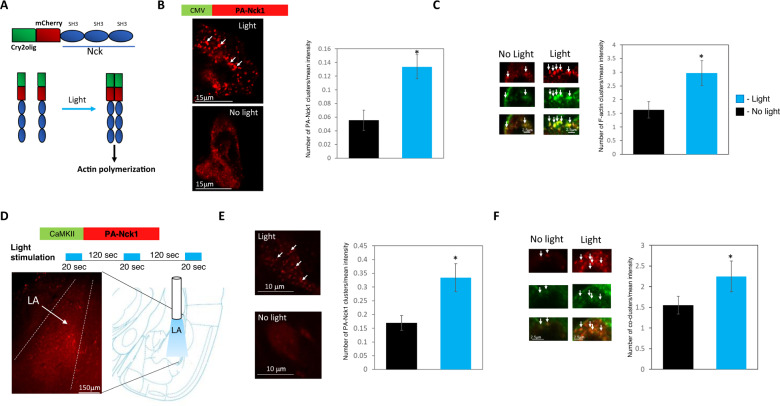
Blue light stimulation of HEK293 cells and lateral amygdala neurons leads to the formation of PA-Nck1 and F-actin clusters. **A** PA-Nck1 contains the 3 SH3 domains of Nck1 conjugated to Cry2olig and mCherry. Blue light stimulation leads to PA-Nck1 clustering. Studies in culture cells have shown that such clustering leads to actin polymerization. **B** HEK293 cells were transfected with PA-Nck1 under the control of the CMV promoter. Stimulation of the cells with light leads to a significant increase in PA-Nck1 clusters (*t*_(22)_ = 3.367, *p* = 0.003) (normalized to fluorescence intensity) when compared with cells that were kept in a dark condition. Representative cells that were transfected with PA-Nck1 and activated by light or remained in the dark are shown. **C** PA-Nck1 is co-localized with F-actin in multiple spots (see arrows) in light stimulation HEK293 cells. F-actin clusters are increased (normalized to fluorescence intensity) significantly after light stimulation (*n* = 7) compared to cells stored in dark (*n* = 6) (*t*_(11)_ = 2.367, *p* = 0.036). **D** Mice were microinjected with AAV expressing the PA-Nck1 under the control of the short CaMKII promoter into the lateral amygdala (LA) and stimulated with blue light through optic fibers. PA-Nck1 construct and light stimulating protocols are shown. Representative expression of PA-Nck1 in LA as observed by mCherry reporter protein expression is shown. **E** Stimulation of LA neurons with light leads to a significant increase (*t*_(28)_ = 2.744, *p* = 0.01) in PA-Nck1 clusters (normalized to fluorescence intensity) in LA compare with neurons that were not stimulated with light. Representative LA neurons that express PA-Nck1 and are activated by light or remain in the dark are shown (single neurons for dark and light conditions are shown). **F** PA-Nck1 is co-localized with F-actin in multiple spots (see arrows) in the light-activated neurons in LA. Application of light to LA leads to a significant increase in PA-Nck1 and F-actin co-localization (*t*_(18)_ = 2.158, *p* = 0.045). Values are mean ± SEM. **B**, **C**, **E**, **F** Independent samples *t*-test.

To further examine whether Nck1 suppresses long-term fear conditioning memory we activated Nck1 in excitatory neurons in LA and examined the effects on long-term fear conditioning memory. To activate the Nck1 protein at a high spatiotemporal resolution we used a novel optogenetic approach. We utilize the CRY2olig–mCh–Nck1 (Fig. 2A) where three SH3 domains of Nck1 are fused to CRY2olig and mCherry to generate a photoactivatable Nck1 (PA-Nck1) that clusters in response to blue light application [34]. We first tested the functionality of PA-Nck1 in HEK293 cells. We transfected HEK293 cells with PA-Nck1 under the control of CMV promoter. We applied blue light (473 nm) and monitored the number of PA-Nck1 clusters (normalized to fluorescence intensity). We detected an increase in PA-Nck1 clusters after blue light stimulation (*n* = 12 cells) compared to cells stored in the dark (*n* = 12 cells) (*t*_(22)_ = 3.367, *p* = 0.003) (Fig. 2B). It was shown that Nck clustering leads to localized actin polymerization [25]. Moreover, actin is co-localized with CRY2olig–mCh–Nck clusters in COS-7 [34]. We stimulated HEK293 cells as above and stained it with the F-actin binding compound phalloidin. As seen in Fig. 2C PA-Nck1 is co-localized with F-actin in multiple spots in light stimulated HEK293 cells. Moreover, we detected an increase in F-actin clusters (normalized to fluorescence intensity) after blue light stimulation (*n* = 7 cells) compared to cells stored in the dark (*n* = 6 cells) (*t*_(11)_ = 2.367, *p* = 0.036) (Fig. 2C). Next, we examined whether PA-Nck1 can form clusters after blue light stimulation in pyramidal neurons in lateral amygdala (LA). We, therefore, cloned PA-Nck1 downstream to short CaMKII promoter in AAV to express it in excitatory pyramidal neurons in LA [35]. PA-Nck1 is expressed in LA after AAV injection (Fig. 2D). We applied blue light (473 nm) on the LA through optic fiber using the same light intensity and duration used in the behavioral paradigm. The brains were removed at the end of the last light stimulation. Light stimulation led to a significant increase in PA-Nck1 clusters (normalized to fluorescence intensity) in LA neurons (*n* = 14 neurons from 8 animals) compared to no-stimulation neurons (*n* = 14 neurons from 6 animals) (*t*_(28)_ = 2.744, *p* = 0.01) (Fig. 2E). Thus, the application of blue light leads to a rapid increase in PA-Nck1 clusters in LA. We next studied whether the increase in PA-Nck1 clusters leads to an increase in clusters where PA-Nck1 is co-localized with F-actin. We revealed that application of light to LA leads to a significant increase in PA-Nck1 and F-actin co-localization in light treated neurons (*n* = 10 neurons from 5 animals) compared to no light neurons (*n* = 10 neurons from 4 animals) (*t*_(18)_ = 2.158, *p* = 0.045) (Fig. 2F).

**Fig. 3:**
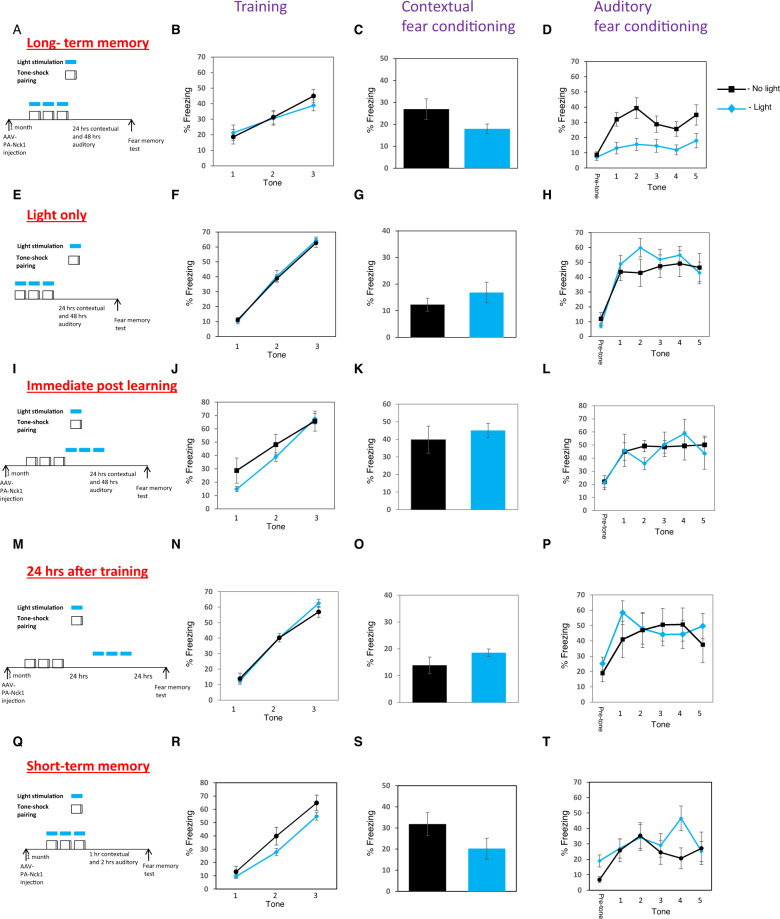
Activation of Nck1 in LA during fear conditioning impaired auditory long-term, but not short-term, fear memory. **A** Description of the behavioral protocol. Mice injected with AAV containing PA-Nck1 into LA were subjected to 3 tone-shock pairings. During each pairing, the animals received blue light (473 nm) illumination in LA to activate the PA-Nck1. Controls were animals injected with AAV, containing the PA-Nck1, into LA, implanted with optic fibers and subjected to fear conditioning but without light. Fear memory was tested 24 h and 48 h after training for contextual and auditory long-term fear conditioning memory (LTM), respectively. **B** Freezing during training in response to the tone CS is not different between light (*n* = 12) and no light (*n* = 13) animals (*F*_(1,21)_ = 0.138, *p* = 0.714). **C** Contextual fear conditioning tested 24 h after fear conditioning is not different between the light and no light groups (*t*_(23)_ = 1.938, *p* = 0.065). **D** Auditory fear conditioning tested 48 h after fear conditioning is impaired in animals subjected to light activation of PA-Nck1 compared to no light mice (*F*_(1,21)_ = 10.369, *p* < 0.005). **E** Animals were subjected to light only (no PA-Nck1 expressed). **F** Freezing throughout the tones presentations during training of animals that were not injected with the virus but subjected to light (light only) or no light control (no light only) that were used in the LTM experiments was analyzed. There is no significant difference between light (*n* = 7) and no light (*n* = 7) groups (*F*_(1,12)_ = 0.109, *p* = 0.747). **G** Contextual fear conditioning tested 24 h after fear conditioning is not different between the light and no light groups (*t*_(12)_ = 0.992, *p* = 0.341). **H** There is no significant difference between the animals that were subjected to light and not light in the long-term auditory fear conditioning memory test (*F*_(1,12)_ = 0.383, *p* = 0.548). **I** Schematic description of the protocol of behavior and light stimulation for the immediate post-training activation of PA-Nck1 experiment. **J** Freezing during training in response to the tone CS is not different between light (*n* = 6) and no light (*n* = 6) animals (*F*_(1,10)_ = 1.899, *p* = 0.198). **K** Contextual fear conditioning tested 24 h after fear conditioning is not different between the light and no light groups (*t*_(10)_ = 0.601, *p* = 0.561). **L** Auditory fear conditioning tested 48 h after fear conditioning is not different between animals subjected to light activation of PA-Nck1 compared to no light mice (*F*_(1,10)_ = 0.026, *p* = 0.875). **M** Schematic description of the protocol of behavior and light stimulation a day after training. **N** Freezing throughout the tones presentations during training of animals used in the experiment. There is no significant difference between light and no light groups (*F*_(1,13)_ = 0.042, *p* = 0.841). **O** Contextual fear conditioning tested 1 day after light stimulation is not different between the light and no light groups (*t*_(13)_ = 1.458, *p* = 0.169). **P** Auditory fear conditioning tested a day after light stimulation is not different between animals subjected to light activation of PA-Nck1 compared to no light mice (*F*_(1,13)_ = 0.097, *p* = 0.76). **Q** Schematic description of the protocol of behavior and light stimulation for the STM experiment. **R** Freezing throughout the tones presentations during training of animals used in the STM experiment subjected to light or no light control was analyzed. There is no significant difference between light and no light groups (*F*_(1,11)_ = 3.372, *p* = 0.093). **S** Contextual fear conditioning tested 1 h after fear conditioning is not different between the light and no light groups (*t*_(11)_ = 1.631, *p* = 0.131). **T** Auditory fear conditioning tested 2 h after fear conditioning is not different between animals subjected to light activation of PA-Nck1 compared to no light mice (*F*_(1,11)_ = 0.673, *p* = 0.43). Values are mean ± SEM. **C**, **G**, **K**, **O**, **S** Independent samples *t*-test. **B**, **D**, **F**, **H**, **J**, **L**, **N**, **P**, **R**, **T** Repeated measures ANOVA.

We next expressed PA-Nck1 in excitatory neurons in LA of mice and applied blue light during tone (CS)-shock (US) presentations (Fig. 3A). There is no effect on fear conditioning training (*F*_(1,21)_ = 0.138, *p* = 0.714) and treatment × tone trial interaction (*F*_(1.588,33.343)_ = 0.534, *p* = 0.551) (Fig. 3B). Next, the mice were tested for contextual fear conditioning LTM 24 h after training. There is no significant difference between mice that were subjected to light stimulation to activate PA-Nck1 (*n* = 12) and mice that were not exposed to light (*n* = 13) (*t*_(23)_ = 1.938, *p* = 0.065) (Fig. 3C). These mice were tested for auditory fear memory 24 h afterward. Long-term auditory fear conditioning is impaired in animals that were stimulated by light compared with animals that were not exposed to light (*F*_(1,21)_ = 10.369, *p* < 0.005) (Fig. 3D). There is no treatment × tone trial interaction (*F*_(4,84)_ = 0.743, *p* = 0.565). Applying light alone (*n* = 7) (Fig. 3E) had no effect on training (*F*_(1,12)_ = 0.109, *p* = 0.747; no interaction *F*_(2,24)_ = 0.162, *p* = 0.852) when compare to the no-light group (*n* = 7) (Fig. 3F). These animals were not different in long-term contextual fear conditioning (*t*_(12)_ = 0.992, *p* = 0.341) (Fig. 3G) and in long-term auditory fear conditioning (*F*_(1,12)_ = 0.383, *p* = 0.548 with no interaction *F*_(4,48)_ = 1.714, *p* = 0.162) (Fig. 3H). Thus, light application per se into LA does not affect long-term fear memory. Taken together, our results show that activation of Nck1 in the LA impairs long-term auditory, but not contextual, fear conditioning memory.

### Activation of Nck1 immediately after fear conditioning training does not affect long-term fear memory formation

We next examined the time window of involvement of Nck1 in fear memory and activated PA-Nck1 immediately after fear conditioning. We trained the animals, that expressed PA-Nck1 in LA, for fear conditioning and shined light through the optic fibers onto LA after the last CS (three pulses of 20-s light similar to the light stimulation protocol performed during training above) (Fig. 3I). Freezing during training was not different between the groups (*F*_(1,10)_ = 1.899, *p* = 0.198) and there is no treatment × tone trial interaction (*F*_(1.085, 10.849)_ = 0.849, *p* = 0.386) (Fig. 3J). Animals were tested for contextual long-term fear memory formation 24 h after training. Post-training light stimulated mice (*n* = 6) were not significantly different in long-term contextual fear conditioning memory when compared to non-stimulated animals (*n* = 6) (*t*_(10)_ = 0.601, *p* = 0.561) (Fig. 3K). There is also no effect on long-term auditory fear memory (*F*_(1,10)_ = 0.026, *p* = 0.875) with no treatment × tone interaction (*F*_(4,40)_ = 1.014, *p* = 0.412) (Fig. 3L). These results together with the aforementioned observations show that Nck1 impairs LTM when activated during training, but not immediately afterward.

### Activation of Nck1 in lateral amygdala does not affect the maintenance of long-term fear conditioning memory

The aforementioned results show that activation of Nck1 in LA during training impairs the ability to consolidate long-term fear memory. We were interested to examine the effects of Nck1 activation on memory after its consolidation. Toward that end, we activated Nck1 a day after fear conditioning and tested fear memory 1 day afterward to examine its roles in the maintenance of long-term memory (Fig. 3M). Freezing during fear conditioning training is not different between the groups (*F*_(1,13)_ = 0.042, *p* = 0.841) with treatment × tone interaction (*F*_(2,26)_ = 3.571, *p* = 0.043) (Fig. 3N). Activation of PA-Nck1 did not affect long-term contextual fear conditioning memory tested between animals that were subjected to light (*n* = 8) and animals that were not subjected to light (*n* = 7) (*t*_(13)_ = 1.458, *p* = 0.169) (Fig. 3O). Activation of PA-Nck1 did not affect auditory long-term fear memory (*F*_(1,13)_ = 0.097, *p* = 0.76) and there is no treatment × tone interaction (*F*_(4,52)_ = 1.463, *p* = 0.227) (Fig. 3P). We, therefore, conclude that activation of Nck1 after fear memory consolidation does not affect the maintenance of long-term memory.

### Activation of Nck1 in lateral amygdala does not affect short-term auditory and contextual fear conditioning memories

To further elucidate whether activation of Nck1 during learning has an effect on the ability of the animal to associate the CS and the US or to consolidate the association into long-term memory we tested the effect of PA-Nck1 activation on short-term memory (STM) formation. Toward that end, PA-Nck1 in excitatory neurons in LA of mice was activated during tone (CS)-shock (US) presentations (Fig. 3Q). STM of contextual and auditory fear memory was tested 1 h and 2 h after training, respectively. There is no difference in freezing to the tone during learning between the groups (*F*_(1,11)_ = 3.372, *p* = 0.093) and no treatment × tone interaction (*F*_(2,22)_ = 1.070, *p* = 0.36) (Fig. 3R). There is no difference in contextual STM between animals where PA-Nck1 was activated (*n* = 7) and the group with no activation (*n* = 6) (*t*_(11)_ = 1.631, *p* = 0.131) (Fig. 3S). There is no different in auditory fear STM between animals where PA-Nck1 was activated (*n* = 7) and the group with no activation (*n* = 6) (*F*_(1,11)_ = 0.673, *p* = 0.43) and no treatment × tone interaction (*F*_(2.048, 22.526)_ = 1.450, *p* = 0.234) (Fig. 3T). Cumulatively, the aforementioned results show that activation of Nck1 in LA during fear conditioning does not affect brain faculties needed for the association of the CS and US but rather on the ability to consolidate the memory into LTM.

### Nck1 is localized to presynapses in lateral amygdala and its activation reduces the frequency of mEPSCs

**Fig. 4:**
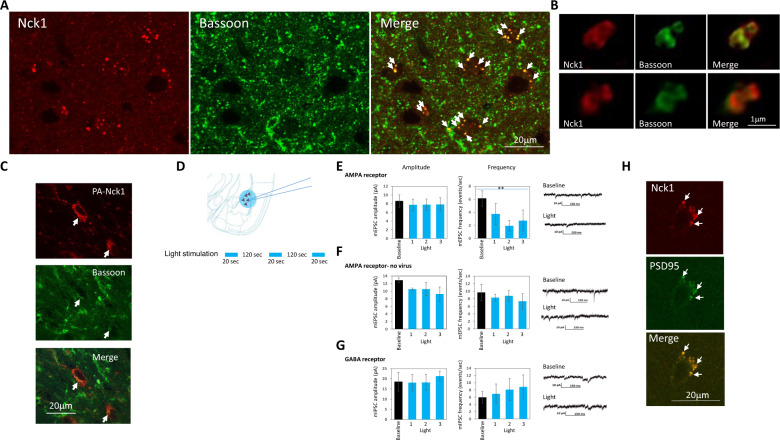
Nck1 is localized to presynapses in LA and its activation decreases the frequency of AMPAR-mediated mEPSCs. **A** Lateral amygdala neurons were co-labeled with antibodies that bind to Nck1 and Bassoon (a presynaptic protein). Nck1 is colocalized with bassoon in presynapse. **B** Super-resolution microscopy reveals that Nck1 localization is almost perfectly aligned with Bassoon in presynapse. **C** PA-Nck1 is co-localized with Bassoon. Arrows show PA-Nck1, Bassoon and the colocalized PA-Nck1 and Bassoon. **D** Schematic description of the voltage patch-clamp preparation and mode of light stimulation. **E** AMPAR-mediated mEPSCs amplitude and frequency before lights stimulation (Baseline; black) and during the 3 light stimuli (blue) are shown. No significant differences in amplitude between the baseline and light stimuli were detected (*F*_(3,9)_ = 1.145, *p* = 0.382). Significant differences were detected in frequencies of mEPSCs between the baseline and light stimuli (*F*_(3,9)_ = 6.642, *p* = 0.012; *n* = 4). **F** Shining light alone on the lateral amygdala that does not express PA-Nck1 does not affect AMPA-mediated mEPSCs (*n* = 3) amplitude (*F*_(1.181,2.362)_ = 2.351, *p* = 0.254) and frequency (*F*_(1.089,2.178)_ = 0.804, *p* = 0.47). **G** GABAR-mediated mIPSCs amplitude and frequency before lights stimulation (Baseline; black) and during the 3 light stimuli (blue). No significant differences were detected (*n* = 4) (amplitude- *F*_(3,9)_ = 0.705, *p* = 0.573; frequency- *F*_(3,9)_ = 0.923, *p* = 0.468). Representative event traces of AMPAR-mediated mEPSCs and GABAR-mediated mIPSCs from neurons recorded in the LA are shown. **H** Lateral amygdala neurons were co-labeled with antibodies that bind to Nck1 and PSD-95. Nck1 is colocalized with PSD-95. Arrows show Nck1, PSD-95 and the colocalized Nck1 and PSD-95. Values are mean ± SEM. **E**–**G** Repeated measure ANOVA with LSD post hoc analysis.

The aforementioned results have shown that Nck1 activation during fear conditioning impairs long-term fear memory formation. However, activation of Nck1 immediately after fear conditioning did not affect long-term fear conditioning memory. This observation suggests that Nck1 affects synaptic transmission during training. To explore such a possibility, we were interested to monitor whether Nck1 is localized near the synapse in areas where it can affect transmission. Indeed, we detected that Nck1 is located to presynapses in LA neurons. Nck1 is colocalized with the presynaptic protein marker bassoon (Fig. 4A). Super-resolution microscopy further revealed that Nck1 is almost perfectly aligned with bassoon (Fig. 4B). Next we were interested to explore whether PA-Nck1 is found at pre-synapse. Figure 4C shows that PA-Nck1 is co-localized with Bassoon indicating that in PA-Nck1 can be found in the presynapse.

To further assess the synaptic mechanisms whereby Nck1 may affect fear memory formation we performed whole-cell voltage-clamp recordings to measure miniature AMPAR-mediated excitatory synaptic currents (mEPSCs) or miniature GABAR-mediated inhibitory synaptic currents (mIPSCs) from LA principal neurons expressing PA-Nck1 in brain slices. We shined blue light (same protocol as behavior) onto LA cells and measured the mEPSCs and mIPSCs during light stimuli and compared it to basal level before stimulation (Fig. 4D). We revealed that activation of PA-Nck1 leads to a reduction of frequency (*F*_(3,9)_ = 6.642, *p* = 0.012; baseline vs. light 1- *p* = 0.022; baseline vs. light 2- *p* = 0.004 baseline light 3 *p* = 0.095) and an increase in rise time (*F*_(3,9)_ = 7.307; *p* = 0.009; baseline vs. light 1 *p* = 0.056; baseline vs. light 2 = 0.01; baseline vs. light 3 = 0.025), but had no effect on amplitude of mEPSCs (*F*_(3,9)_ = 1.145, *p* = 0.382), of the AMPAR-mediated mEPSCs (Fig. 4E) (*n* = 4 cells from 4 animals; 1 cell per brain slice). We examined whether light per se affects AMPA-mediated mEPSCs frequency and AMPAR rise time. We found no effect on amplitude (*F*_(1.181,2.362)_ = 2.351, *p* = 0.254), frequency (*F*_(1.089,2.178)_ = 0.804, *p* = 0.47) and rise time (*F*_(1.555,3.110)_ = 1.127, *p* = 0.407) (Fig. 4F) (*n* = 3 cells *n* = 3 animals; 1 cell per brain slice). Application of light on LA and activation of PA-Nck1 has no effect on GABAR-mediated mIPSCs (amplitude- *F*_(3,9)_ = 0.705, *p* = 0.573; frequency- *F*_(3,9)_ = 0.923, *p* = 0.468; rise time- *F*_(3,9)_ = 0.427, *p* = 0.738) of PA-Nck1 expressing neurons (Fig. 4G) (*n* = 4 cells from an animal; 1 cell per brain slice). Cumulatively, these results show that Nck1 is localized to the presynapse and that activation of Nck1 reduces the probability of presynaptic glutamate release. However, we also detected that Nck1 is co-localized with PSD-95 (Fig. 4H) and cannot exclude a postsynaptic role of Nck1 in memory formation.

## Discussion

Adaptor proteins are key proteins in neurons that mediate the transfer of information from receptors activated after synaptic stimulation into intracellular signaling. Nck1 is a central protein that transfers information from membrane receptors to actin cytoskeleton to induce cellular events [23, 24]. Studies have shown that Nck1 activity affects actin cytoskeleton polymerization [26–29]. Actin cytoskeleton polymerization is involved in key neuronal functions such as morphogenesis and transmission [18–20] that are believed to be needed for memory formation. In this study, we were therefore interested to examine whether Nck1 protein is involved in fear memory formation. Toward that end, we decreased Nck1 levels by shRNA in lateral amygdala (LA), a brain region needed for fear memory formation. Reduction of Nck1 in LA enhanced long-term, but not short-term, auditory fear conditioning memory. This result shows that Nck1 restricts neuronal processes that are needed for the consolidation of long-term memory. Activation of PA-Nck1 by light in LA during fear conditioning training impaired long-term, but not short-term, auditory fear conditioning memory. These observations show that the increase of Nck1 activity during training does not affect auditory fear memory acquisition but rather its consolidation into long-term memory. Thus, Nck1 serves to modulate the ability of amygdala neurons to form long-term memory.

We also found that activation of Nck1 in LA during fear conditioning training, but not immediately afterward, impairs long-term auditory fear conditioning memory. This observation implies that Nck1 activity in LA may restrict the formation of long-term fear memory by affecting synaptic transmission. This assumption was further strengthened by our finding that Nck1 is localized to the presynapse. We, therefore, examined the effect of Nck1 activity on AMPA receptor-mediated mEPSCs or GABA receptor-mediated mIPSCs. We found that activation of PA-Nck1 in LA led to a decrease in the frequency of AMPAR-mediated mEPSCs. GABAR-mediated mIPSCs were not affected by the activation. The reduction in mEPSCs frequency indicates a decrease in the probability of synaptic glutamate release [37]. Thus, Nck1 activation restricts glutamate release an event that affects fear memory formation. For example, it was shown that AMPA receptors are essential for fear memory formation [38]. Thus, reduction in glutamate release by Nck1 affects fear conditioning and restricts long-term fear memory. Interestingly, the reduction in presynaptic glutamate release is not sufficient to affect short-term memory formation. Therefore, Nck1 activity provides a threshold specifically for long-term fear memory formation by restricting glutamate release in LA.

It is plausible that Nck1 activation mediates long-lasting neuronal alterations involved in memory consolidation in LA, rather than transient events involved in memory acquisition and short-term memory. It could be, for example, that glutamate release mediates long-lasting postsynaptic structural changes. Ample studies show that glutamate can affect neuronal structural changes such as spine morphogenesis [39]. Lasting changes in the number and shape of dendritic spines were observed following learning [17]. Alternatively, it could be that Nck1 activation initiates changes in the presynapse that inhibit neurotransmitter release that can last for a long period of time [40]. Such lasting pre- and post-synaptic alterations may not affect STM that can be dependent on other cellular events such as transient protein modifications (e.g., phosphorylation).

It was shown that Latrunculin A (Lat-A), which promotes actin depolymerization by sequestering actin monomers [41] increased the frequency of mEPSC by ∼5 fold in cultured hippocampal neurons [42] and addition of Cytochalasin D, that binds to F-actin polymer and prevents polymerization, also caused an increase in the rate of mEPSCs from CA1 pyramidal neurons [43]. It is further shown that in the presence of Lat-A, the speed of exocytosis from axonal terminals is faster [44]. Thus, actin polymers restrain transmitters release. We show here that activation of Nck1 inhibits synaptic release possibly by forming an actin cytoskeletal restraining structure.

Imbalanced in actin polymerization and its regulatory proteins are associated with cognitive dysfunctions and are involved in mental brain diseases including, autism spectrum disorders (ASDs), schizophrenia, and intellectual disability [21]. Some of the proteins that are involved in such abnormalities (e.g., NCKAP1, Arp2/3, WAVE) are associated with Nck1 pathways. Moreover, Nck1 is associated with brain dysfunctions [30–32]. However, its possible effects on memory were not known. Our results show that Nck1 activation regulates long-term fear memory formation. Thus, Nck1 serves to adjust the formation of the appropriate fear memory for the right response to a fearful experience. It would be interesting to further examine whether the level of Nck1 is different in patients that suffer from phobias or that experienced a traumatic event that leads to an increased long-term fear response. In these cases, Nck1 level or activity might be reduced compared to normal people. Moreover, some individuals are more resilient to traumatic events and it would be important to examine whether Nck1 level or activity is increased in these individuals.
